# Clinical utility of proenkephalin A 119-159 for prediction of worsening renal function and prognosis in patients with sepsis –results of a patient-level meta-analysis

**DOI:** 10.1186/s13054-026-05947-5

**Published:** 2026-03-24

**Authors:** Birte Arlt, Pietro Caironi, Jennifer Meessen, Oliver Hartmann, Alexandre Mebazaa, Matthieu Legrand, Etienne Gayat, Christian Nusshag, Thorsten Brenner, Pierre François Laterre, Ornella Tinelli, Roberto Latini, Florian Uhle

**Affiliations:** 1 SphingoTec GmbH, Hennigsdorf, Germany; 2 Department of Oncology, University of Turin, Turin, Italy; 3 Department of Acute, Brain and Cardiovascular Injury, Institute for Pharmacological Research Mario Negri IRCCS, Milan, Italy; 4 4Teen4 Pharmaceuticals GmbH, Hennigsdorf, Germany; 5 Department of Anaesthesiology and Critical Care Medicine, Assistance Publique-Hôpitaux de Paris, Saint Louis and Lariboisière University Hospitals, Paris, France; 6 Department of Anesthesia and Perioperative Care, Division of Critical Care Medicine, University of California, San Francisco, San Francisco, CA USA; 7 Department of Nephrology, Heidelberg University Hospital, Heidelberg University, Heidelberg, Germany; 8 Department of Anesthesiology and Intensive Care Medicine, University Hospital Essen, University Duisburg-Essen, Essen, Germany; 9 Department of Intensive Care, Saint Luc University Hospital, Brussels, Belgium

**Keywords:** Sepsis, Septic shock, Proenkephalin A, Renal function, Biomarker

## Abstract

**Background:**

Proenkephalin A 119–159 (penKid) is a functional kidney biomarker inversely correlating with the glomerular filtration rate. We conducted a patient-level meta-analysis to evaluate whether penKid provides additional value to serum creatinine (sCr) for assessing kidney function and outcome in patients with sepsis or septic shock.

**Methods:**

To identify eligible studies, the database PubMed and internal resources have been searched in March 2025. Randomized controlled trials or observational trials enrolling patients with sepsis or septic shock admitted to ICU (> 100 patients per study) were included. The primary endpoint was worsening renal function (WRF) defined as a further increase of sCr equal or greater 0.3 mg/dL within 48 h from ICU admission value (baseline), or need for renal replacement therapy (RRT) or death within 48 h. Secondary endpoint was 28d-mortality. Subgroups were created applying upper normal reference values for penKid and sCr.

**Results:**

Individual data from 2,203 patients with sepsis or septic shock enrolled in four studies were included. penKid was independently associated with WRF (OR 3.4, 95% CI 2.7–4.3, *p* < 0.0001) and 28d-mortality (OR 1.6, 95% CI 1.4–1.9, *p* < 0.0001). Among patients with normal sCr at admission, those with elevated penKid had a higher incidence of WRF compared to patients with normal penKid (27.1% vs. 11.1%, *p* < 0.0001) and the highest 28d-mortality (38.6% vs. 15.3%, *p* < 0.0001).

**Conclusion:**

penKid adds predictive value beyond sCr for identifying patients at risk for WRF and 28d-mortality. Importantly, elevated penKid identifies at-risk patients even when sCr is normal. Combining both biomarkers may therefore offer a valuable tool for risk stratification in patients with sepsis or septic shock.

**Trial registration:**

This meta-analysis has been retrospectively registered at PROSPERO, CRD420251153702. Registered 23 September 2025.

**Supplementary Information:**

The online version contains supplementary material available at 10.1186/s13054-026-05947-5.

## Background

Acute kidney injury (AKI) is characterized by a rapid decline in kidney function and is associated with high morbidity and mortality [1]. Among critically ill patients, sepsis accounts for approximately 45–70% of all AKI cases [2]. It is common practice to use serum creatinine (sCr) and urine output for diagnosing and monitoring renal (dys)function, based on the current KDIGO definition of AKI [3]. However, the inherent limitations of these criteria urge the need for additional biomarkers to enable an informed decision-making process.

Recent data demonstrate that various functional and damage biomarkers have clinical utility to improve diagnosis and prediction of risk for renal dysfunction alone or side-by-side with the current criteria [4–9]. Proenkephalin A 119–159 (penKid) is a functional biomarker that identifies patients with sepsis who are at an increased risk of developing AKI and major adverse kidney events [10, 11]. In septic patients admitted to ICUs, penKid predicts AKI within 48 h and rises progressively with the severity of AKI [12].

Even though penKid has shown promise in enhancing early diagnosis, prediction and risk stratification of renal dysfunction, the evidence base supporting the combined clinical utility of penKid and sCr remains scarce. Available studies vary in their inclusion criteria, and endpoint definitions of renal dysfunction, limiting statistical power and precluding robust subgroup analyses. To overcome current evidence limitations, we aimed to conduct a patient-level meta-analysis allowing for a harmonized endpoint definition of worsening renal function across datasets and the assessment of a combined clinical performance of penKid and sCr in patients with sepsis or septic shock. By leveraging patient-level data from multiple cohorts, our patient-level meta-analysis aims to generate clinically actionable insights into the utility of biomarker-guided risk assessment in sepsis-associated AKI.

## Methods

### Search strategy and study selection

This study was conducted according to the PRISMA guidelines (the checklist and further methodology is available in the supplement) [13]. Studies were included when the following criteria were fulfilled: (i) randomized controlled trials or observational trials and (ii) patients with sepsis or septic shock admitted to ICU (> 100 patients per study). Risk of bias in individual studies included in this meta-analysis have been assessed using the QUADAS-2 tool.

### Endpoint definition

The primary endpoint was worsening renal function (WRF) defined as a further increase of sCr equal or greater 0.3 mg/dL within 48 h from ICU admission value (baseline), or need for renal replacement therapy (RRT) or death within 48 h. The secondary endpoint was 28d all-cause mortality. The primary endpoint (WRF) was adjusted for age, sex, sCr, chronic kidney disease (CKD), hypertension, SAPS II score, lactate and septic shock. The secondary endpoint (28d-mortality) was adjusted for age, sex, CKD, SAPS II score, lactate and presence of septic shock.

### Statistical methods

All analyses have been conducted using a one-stage individual patient data meta-analytic framework, incorporating data from all patients across studies into a single model. Values are expressed as medians [interquartile ranges (IQR)] or counts (percentages) as appropriate. For penKid the predefined cut-off at 89 pmol/L was applied [11]; for sCr, a sex-specific cut point − 1.22 mg/dL for males and 1.01 mg/dL for females - was applied [14]. For both endpoints, incidence rates are calculated. For multivariable analysis, binary logistic regression was performed after adjustment of potential confounders. To jointly evaluate both biomarkers, patients were stratified into four subgroups, based on their sCr and penKid levels. Patients with normal penKid and normal sCr served as the reference group.

## Results

Four clinical studies met the eligibility criteria and were included [12, 15–17]. The study selection process, including reasons for exclusion, is shown in Suppl. Figure 1 and Suppl. Table 9, and detailed study characteristics and methodological quality are presented in Suppl. Tables 1 and 2.

In total, 2,203 patients were analyzed (Suppl. Figure 2). Median age was 68 years [57–77], and 61.8% were male. Overall, 22.6% required RRT, 11.8% had CKD, 35.1% had sepsis, and 64.9% had septic shock. Median SOFA and SAPS scores were 8 [range 5–11] and 50 [range 38–63], respectively **(**Table 1**)**. Patient characteristics by biomarker subgroups and by study are provided in Suppl. Tables 3–6.

**Table 1:** Patient characteristics at admission and during ICU stay

	*n*	All	No WRF (*n* = 1559, 70.8%)	WRF (*n* = 644, 29.2%)	*P*-value	28d-survival (*n* = 1651, 74.9%)	28d-mortality (*n* = 552, 25.1%)	*P*-value
Age, median [IQR]	2,203	68 [57–77]	67 [56–76]	69 [60–77]	0.0011	66 [55–75]	73 [63–80]	< 0.0001
Sex (male), no. (%)	2,203	1362 (61.8)	947 (60.7)	415 (64.4)	0.115	1012 (61.3)	350 (63.4)	0.4051
Body mass index in kg/m², median [IQR]	1,967	26.12 [23.44–29.41]	26.03 [23.21–29.40]	26.15 [23.82–29.41]	0.1204	26.12 [23.44–29.57]	26.12 [23.15–29.38]	0.3562
Hypertension, no. (%)	2,019	768 (38)	500 (35.5)	268 (43.8)	0.0005	226 (43)	542 (36.3)	0.0070
Chronic kidney disease, no. (%)	2,032	239 (11.8)	112 (7.9)	127 (21)	< 0.0001	159 (10.6)	80 (15.1)	0.0071
Renal replacement therapy within 48 h, no. (%)	2,203	257 (11.7)	0 (0)	257 (39.9)	< 0.0001	146 (8.8)	111 (20.1)	< 0.0001
Renal replacement therapy > 48 h, no. (%)	2,203	497 (22.6)	136 (8.7)	361 (56.1)	< 0.0001	293 (17.7)	204 (37)	< 0.0001
Sepsis without shock, no. (%)	2,203	774 (35.1)	622 (39.9)	152 (23.6)	< 0.0001	640 (38.8)	134 (24.3)	< 0.0001
Septic Shock, no. (%)	2,203	1429 (64.9)	937 (60.1)	492 (76.4)	< 0.0001	1011 (61.2)	418 (75.7)	< 0.0001
SAPS II score, median [IQR]	2,203	50 [38–63]	46 [36–58]	59 [48–73]	< 0.0001	47 [36–59]	59 [48–72]	< 0.0001
SOFA score, median [IQR]	1,295	8 [5–11]	7 [4–9]	11 [8–13]	< 0.0001	7 [5–10]	10 [7–12]	< 0.0001
Lactate in mmol/L, [IQR]	2,075	1.8 [1.2–2.9]	1.6 [1.1–2.5]	2.4 [1.5–4.6]	< 0.0001	1.6 [1.1–2.6]	2.4 [1.4–4.6]	< 0.0001
Serum creatinine in mg/dL, median [IQR]	2,203	1.30 [0.81–2.28]	1.10 [0.75–1.83]	2.03 [1.30–3.19]	< 0.0001	1.21 [0.80–2.10]	1.64 [1.00-2.60]	< 0.0001
Proenkephalin A in pmol/L, median [IQR]	2,203	82.10 [48.37–152.30]	68.40 [42.69-112.54]	153.66 [81.79-237.61]	< 0.0001	73.77 [44.62-134.02]	115.15 [73.04-204.26]	< 0.0001

### Individual performance of penKid and sCr

WRF occurred in 29.2% of patients; 56.1% required RRT and 21.0% had pre-existing CKD **(**Table 1**)**. Admission penKid and sCr were higher in patients with WRF than in those without (penKid 153.7 [81.8-237.6] pmol/L vs. 68.4 [42.7-112.5] pmol/L; sCr 2.03 [1.30–3.19] mg/dL vs. 1.10 [0.75–1.83] mg/dL; both *p* < 0.0001). PenKid was independently associated with WRF (adjusted OR 3.27, 95% CI 2.52–4.24, *p* < 0.0001).

Overall, 28-day mortality was 25.1%, with 53.8% of non-survivors developing WRF **(**Table 1**)**. Non-survivors had higher admission penKid and sCr (penKid 115.2 [73.0-204.3] vs. 73.8 [44.6–134.0] pmol/L; sCr 1.6 [1.0-2.6] mg/dL vs. 1.2 [0.8–2.1] mg/dL; both *p* < 0.0001). Admission penKid was independently associated with 28-day mortality (adjusted OR 1.64, 95% CI 1.37–1.96, *p* < 0.0001), and Kaplan–Meier analysis showed reduced 28-day survival for elevated penKid (OR 1.81, 95% CI 1.44–2.27, *p* < 0.0001), but not for elevated sCr (OR 1.06, 95% CI 0.84–1.33, *p* = 0.6487) (Suppl. Figure 3).

Incidence rates, receiver-operating characteristics analysis and performance data for each biomarker are shown in the Suppl. Figure 4 and Suppl. Table 7.

### Combinational performance of penKid and sCr

To jointly evaluate both biomarkers, patients were stratified into four subgroups, based on their sCr and penKid levels.

**Fig. 1:**
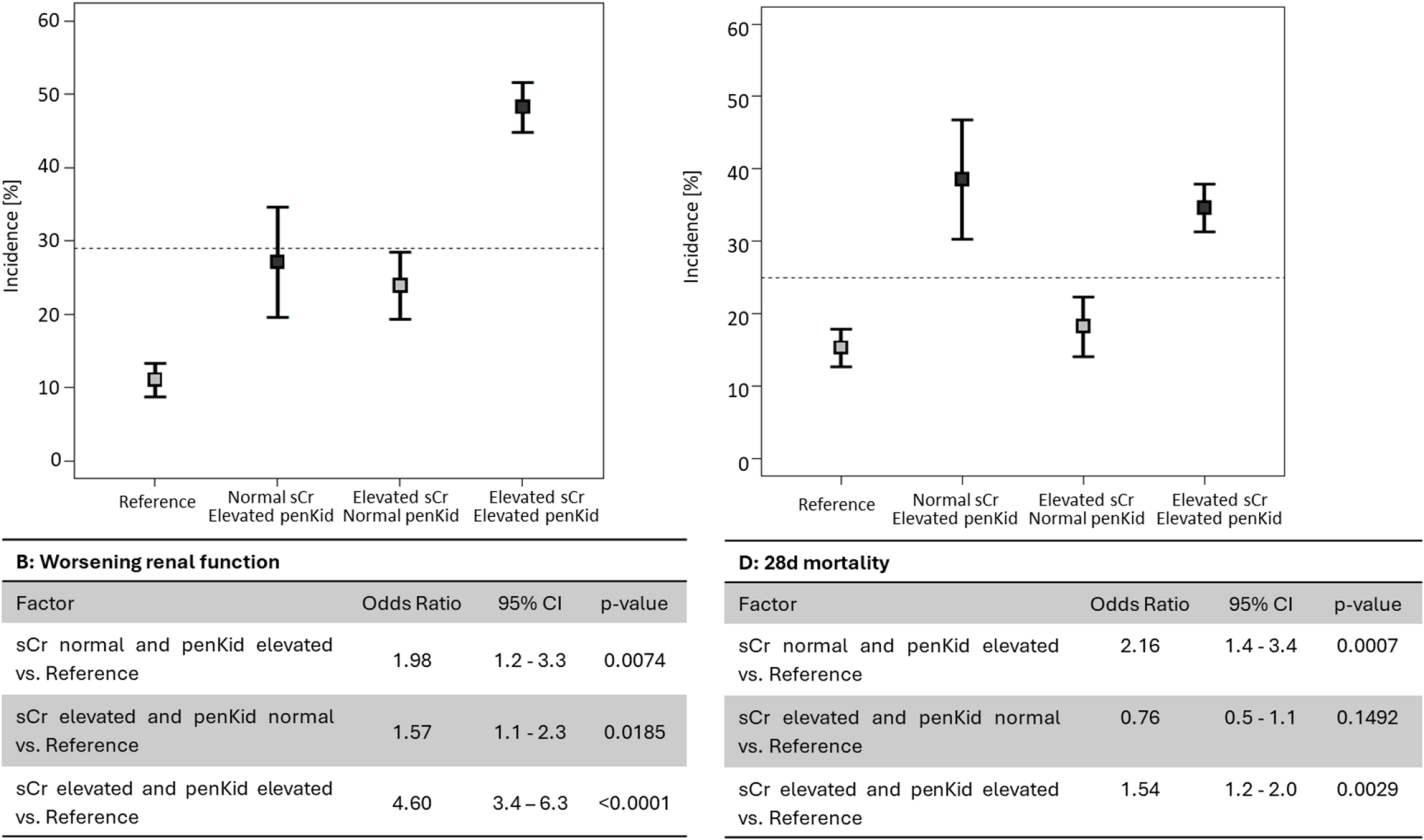
Incidence rates (**A**-**B**) and logistic regression analysis (**C**-**D**) comparing subgroups based on the combination of sCr and penKid (clinical cut-off 89 pmol/L for penKid and a sex-specific cut-off for sCr 1.22 mg/dL for males and 1.01 mg/dL for females) for the primary endpoint WRF (A&C) and the secondary endpoint 28d-mortality (B&D). Odds ratios were adjusted for age, sex, sCr, SAPS II score, lactate, chronic kidney disease, hypertension and septic shock (WRF) and for age, sex, SAPS II score, lactate, chronic kidney disease and presence of septic shock (28d-mortality). Patients with normal penKid and normal sCr served as the reference group. Dashed line represents the pretest probability (WRF: 29.2% *n* = 644, 28d-mortality: 25.1% *n* = 552). penKid – Proenkephalin A 119–159, sCr – serum creatinine

In the reference group, WRF occurred in 11%. With isolated biomarker elevation, WRF increased to 27.1% in patients with elevated penKid (*p* < 0.0001) and to 24.0% in those with elevated creatinine (*p* < 0.0001). The highest incidence was observed in patients with both biomarkers elevated (48.3%; *p* < 0.0001) **(**Fig. 1A**)**. Logistic regression showed an odds ratio of 1.98 (95% CI 1.2–3.3; *p* = 0.0074) for WRF in patients with normal sCr and elevated penKid, exceeding that of single elevated sCr (OR 1.57; 95% CI 1.1–2.3; *p* = 0.0185). In patients with both biomarkers elevated, the odds ratio increased to 4.60 (95% CI 3.4–6.3; *p* < 0.0001) **(**Fig. 1B**)**.

The 28-day mortality rate in the reference group was 15.3%. Elevated penKid alone increased mortality to 38.6% (*p* < 0.0001), similar to combined elevation of both biomarkers (34.7%), whereas isolated elevated sCr remained comparable to reference levels (18.3%; *p* = 0.2360) **(**Fig. 1C**)**. Logistic regression confirmed a higher 28-day mortality risk in patients with elevated penKid (OR 2.16; 95% CI 1.4–3.4; *p* = 0.0007). Isolated elevated sCr showed no increased risk (OR 0.76; 95% CI 0.5–1.1; *p* = 0.1492), whereas combined elevation of both biomarkers increased risk (OR 1.54; 95% CI 1.2–2.0; *p* = 0.0029) **(**Fig. 1D**)**.

**Fig. 2:**
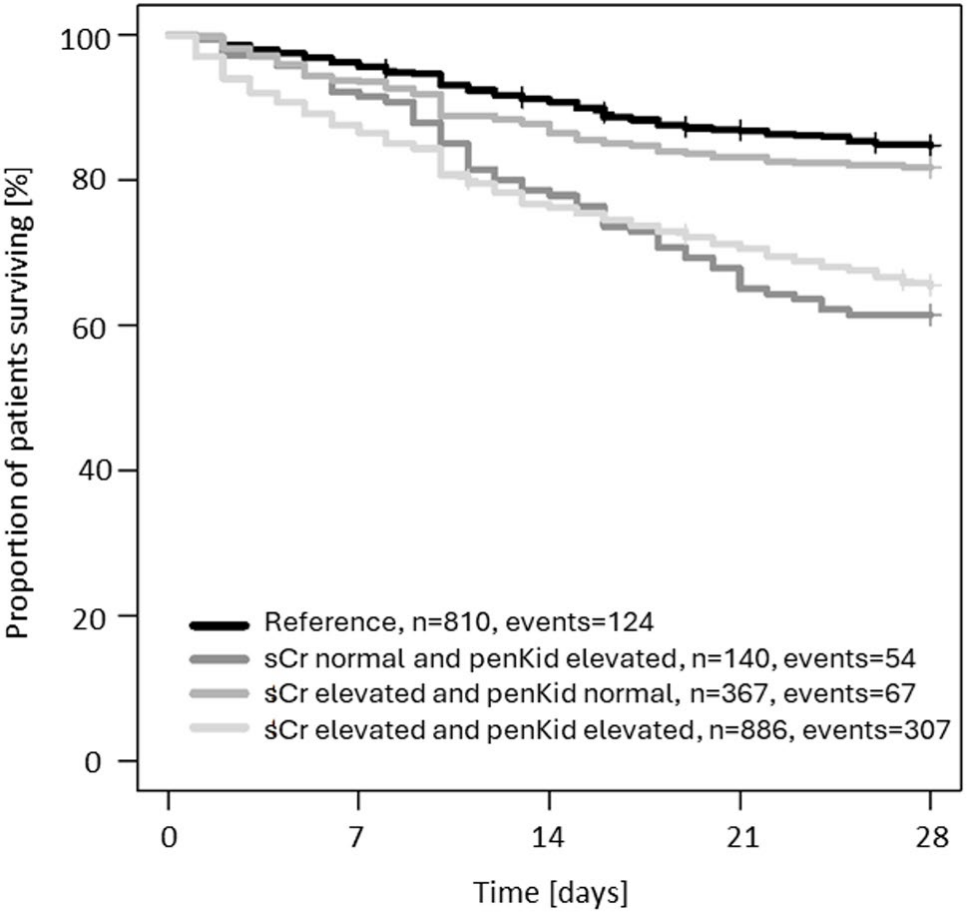
Kaplan Meier survival curves for 28d-mortality comparing subgroups with normal and elevated sCr and penKid based on the clinical cut-off 89 pmol/L for penKid and a sex-specific cut-off for sCr (1.22 mg/dL for males and 1.01 mg/dL for females)

Kaplan Meier analysis demonstrated a markedly lower survival probability over 28 days in patients with normal sCr and elevated penKid, similar to patients with both biomarkers being elevated **(**Fig. 2**)**. Receiver-operating characteristics analysis and performance data of both biomarkers are shown in the Suppl. Figure 5 and Suppl Table 8.

## Discussion

This is the first patient-level meta-analysis assessing the clinical performance of the functional biomarker penKid in addition to the diagnostic standard sCr in a large cohort of critically ill patients with sepsis or septic shock. Patients presenting with physiologically normal sCr but elevated penKid at admission had a moderately elevated risk for WRF while experiencing a significantly elevated risk for 28d-mortality compared to patients with normal penKid values. Twenty-eight-day mortality was as high as in patients admitted with both elevated sCr and penKid.

Previous evidence has demonstrated that using penKid allows for better phenotyping of patients who do not meet the definition of AKI, a stage defined as subclinical AKI (sub-AKI) [6]. Interestingly, patients identified as having sub-AKI exhibited higher 28d-mortality rates compared to patients with no AKI, with mortality rates approaching those of patients with established AKI [6]. Our analysis confirms that penKid identifies septic and septic shock patients at an elevated risk for developing WRF, even when these patients have normal sCr. Importantly, these patients carry a risk profile extending beyond the 48 h time window of WRF, as their 28d-mortality is even higher than in the group of patients with elevated sCr and elevated penKid values upon admission. These high-risk patients are currently masked when clinical assessment is done with sCr alone.

This finding sheds light on the importance of considering more than one functional biomarker for assessing kidney health. Evaluating a “function with function” scenario, where both functional biomarkers are considered in relation to each other, provides added value even though it is not formally covered by the current framework of sub-AKI which was defined as damage versus function by the Acute Disease Quality Initiative [18]. This approach focuses on tracking functional decline, independent from the absence of structural damage, allowing for the early identification of at-risk patients whose kidney function may deteriorate without overt injury, such as patients with heart failure [19]. While sCr has been the gold standard for assessing kidney function, the dynamic nature of penKid might in certain cases enable an earlier detection of kidney function decline before they are apparent with sCr alone. By assessing both biomarkers in parallel, clinicians might overcome limitations and capture more subtle changes in kidney function that might otherwise go undetected by sCr alone, allowing for earlier intervention and potentially improving patient outcomes.

Interestingly, patients with elevated sCr values but normal penKid exhibited an increased risk of developing WRF; however, their 28d-mortality was comparable to that of patients with normal sCr and normal penKid. The marginal increase in 28d-mortality compared to the low-risk group of patients with normal SCr and normal penKid point towards a moderate prognostic risk for these patients as defined by biomarkers. It is conceivable that these patients might already be under recovery at the time of blood sampling, depending on when study blood sampling took place with respect to initiation of early fluid resuscitation and other critical care treatment modalities, which should be addressed in subsequent studies.

The key strength of this meta-analysis is its sample size derived from diverse multicentric studies and a *de novo* patient-level data analysis. Over 2,200 critically ill patients from a total of four clinical trials were included, and kidney function was assessed using a single harmonized and well-established definition of WRF.

Our study, however, has limitations. First, patients were diagnosed with sepsis and septic shock based on various and partly outdated definitions. Second, sCr has been used as a biomarker in this analysis and was also a component of the definition of the primary endpoint WRF, which might bias performance in favor of sCr. False negatives for penKid might be true negatives and findings would need to be confirmed in a sCr-independent, yet valid kidney endpoint which is complex to construct. Third, WRF has been defined based on an increase in sCr referring to the admission value. Further analysis using endpoints such as KDIGO-defined AKI were not feasible due to missing baseline sCr results before admission and the lack of reliable urine output data. Lastly, a meta-analysis may carry some degree of selection bias as well as heterogeneity.

## Conclusion

In this patient-level meta-analysis, penKid has been confirmed as a tool for prediction of WRF and 28d-mortality in patients with sepsis and septic shock. penKid provides additional value beyond sCr, enabling risk stratification at ICU admission for both WRF and 28d-mortality. Combining insights from both functional biomarkers may offer a more differentiated view of kidney health and may support risk mitigation, prevention, and attenuation strategies in this high-risk patient population.

## Supplementary Information

Below is the link to the electronic supplementary material.

Supplementary Material 1

Supplementary Material 2

Supplementary Material 3

## Data Availability

The datasets used and analysed for this meta-analysis are available from the respective principle investigators of the studies on reasonable request.

## Abbreviations

AKI: Acute kidney injury
AUC: Area under the curve
BMI: Body mass index
CKD: Chronic kidney disease
KDIGO: Kidney Disease Improving Global Outcomes
OR: Odds ratio
penKid: Proenkephalin A 119–159
PRISMA: Preferred Reporting Items for Systematic Reviews and Meta–Analysis
QUADAS: 2–Quality Assessment of Diagnostic Accuracy Studies
RRT: Renal replacement therapy
ROC: Receiver–operating–characteristic
SAPS: Simplified Acute Physiology Score
sCr: Serum creatinine
SOFA: Sepsis–related organ failure assessment
Sub-AKI: Subclinical acute kidney injury
WRF: Worsening renal function
