# Supplementary material for: Clinical utility of proenkephalin A 119-159 for prediction of worsening renal function and prognosis in patients with sepsis –results of a patient-level meta-analysis
Source: Crit Care. 2026 Mar 24;30:164. doi: 10.1186/s13054-026-05947-5 (PMC13064392; doi:10.1186/s13054-026-05947-5)
Supplement: Supplementary file 1 — Supplementary Material 1 [file 13054_2026_5947_MOESM1_ESM.docx]

**Supplementary Tables**

**Supplementary Table 1:** Study description.

**Supplementary Table 2:** Quadas-2 risk of bias assessment.

**Supplementary Table 3:** Patient characteristics at baseline grouped by normal and elevated sCr and penKid.

**Supplementary Tabel 4:** Patient characteristics at baseline per study included in the patient-level meta-analysis.

**Supplementary Table 5:** Number of patients per study grouped by the primary endpoint WRF and the secondary endpoint 28d-mortality.

**Supplementary Table 6:** Number of patients per study grouped by normal and elevated sCr and penKid based on the clinical cut-off 89 pmol/L for penKid and a sex-specific cut-off for sCr (1.22 mg/dL for males and 1.01 mg/dL for females).

**Supplementary Table 7A:** Performance characteristics of penKid for the primary endpoint WRF.

**Supplementary Table 7B:** Performance characteristics of penKid for the secondary endpoint 28d-mortality.

**Supplementary Table 8A:** Performance characteristics of penKid in the subgroups normal- and elevated sCr for the primary endpoint WRF.

**Supplementary Table 8B:** Performance characteristics of penKid in the subgroups normal- and elevated sCr for the secondary endpoint 28d-mortality.

***Supplementary Table 9:*** *Literature appraisal indicating title and abstract screening and full-text screening including exclusion rationale.*

Supplementary Table 1: Study description.

| **Study** | **Study design** | **Country of enrollment** | **Patient inclusion criteria** | **Patient exclusion criteria** | **No.of patients enrolled** | **Registration number and reference** |
| --- | --- | --- | --- | --- | --- | --- |
| AdrenOSS-1 (Adrenomedullin and Outcome in Severe Sepsis and Septic Shock) | multicenter, prospective, observational | France, Belgium, Netherlands, Italy, Germany | - Patients admitted to the ICU for sepsis or septic shock (based on the definitions for sepsis and organ failure from 2001 (1) or transferred from another ICU in the state of sepsis and septic shock within less than 24h after admission - If patients were treated with vasopressors, they were considered eligible only if treatment had been started within a maximum of 24 hours after the   primary admission before ICU admission   - Signed informed consent form | - Age <18 years - Pregnancy - Vegetative coma - Participation in another interventional clinical trial in the preceding month | 583 | NCT02393781  (2) |
| FROG-ICU (French and euRopean Outcome registry in Intensive Care Units) | multicenter, prospective, observational | France, Belgium | - Patients at the ICU with a requirement for invasive mechanical ventilation and/or vasoactive drug support for more than 24 h following ICU admission and discharged from ICU - Oral consent to participate | - Age <18 years - Severe head injury (initial Glasgow Coma Scale <8) or brain death or a persistent vegetative state - Pregnancy or breastfeeding - Transplantation in the past 12 months - Not expected to survive or to leave the hospital - No social security coverage | 2,087 | NCT01367093  (3) |
| ALBIOS (Albumin Italian Outcome Sepsis) | Multicenter, randomized | Italy | - Patients with severe sepsis or septic shock (4) within the previous 24h and at any time during the ICU stay | - Age <18 years - Terminal state - Known adverse reaction to albumin administration - Proved or suspected head injury - Congestive heart failure (New York Heart Association class of 3 or 4) - Pathological conditions in which albumin administration is clinically indicated (hepatic cirrhosis with ascites, intestinal malabsorption syndrome, nephrotic syndrome, burns) - More than 24h since inclusion criteria were met - Religious objection to the administration of human blood products - Participation in another experimental study | 1,818 | NCT00707122  (5, 6) |
| PredARRT-Sep | Monocenter, prospective, observational | Germany | - Critically ill patients fulfilling the sepsis-3 criteria at ICU admission (7) - Written informed consent | - Age <18 years - Refusal to participate - Pre-existing RRT dependency or immediate need for RRT at the time of ICU admission - Decompensated liver cirrhosis (hepatorenal syndrome) - No urinary catheter - Life expectancy shorter than 24h | 200 | DRKS00012446  (8, 9) |

Supplementary Table 2: Quadas-2 risk of bias assessment.

Supplementary Table 3: Patient characteristics at baseline grouped by normal and elevated sCr and penKid based on the clinical cut-off 89 pmol/L for penKid and a sex-specific cut-off for sCr (1.22 mg/dL for males and 1.01 mg/dL for females).

|  | **n** | **All** | **Normal SCr, normal penKid (n=810, 36.8%)** | **Normal SCr, elevated penKid (n=140, 6.4%)** | **Elevated SCr, normal penKid (n=367, 16.7%)** | **Elevated SCr, elevated penKid (n=886, 40.2%)** | **P-value** |
| --- | --- | --- | --- | --- | --- | --- | --- |
| Age, median [IQR] | 2203 | 68 [57-77] | 63 [51-73] | 71 [63-78] | 67 [58-76] | 71 [62-79] | <0.0001 |
| Sex (male), no. (%) | 2203 | 1362 (61.8) | 516 (63.7) | 66 (47.1) | 238 (64.9) | 542 (61.2) | 0.0014 |
| Body mass index in kg/m², median [IQR] | 1967 | 26.12 [23.44-29.41] | 25.02 [22.49-28.28] | 24.22 [22.06-28.97] | 27.62 [24.68-31.56] | 26.59 [23.96-30.12] | <0.0001 |
| CKD, no. (%) | 2032 | 239 (11.8) | 12 (1.6) | 11 (8.0) | 27 (8.2) | 189 (22.9) | <0.0001 |
| RRT, no. (%) | 2203 | 497 (22.6) | 59 (7.3) | 27 (19.3) | 64 (17.4) | 347 (39.2) | <0.0001 |
| Sepsis, no. (%) | 2203 | 774 (35.1) | 353 (43.6) | 48 (34.3) | 113 (30.8) | 260 (29.3) | <0.0001 |
| Septic Shock, no. (%) | 2203 | 1429 (64.9) | 457 (56.4) | 92 (65.7) | 254 (69.2) | 626 (70.7) | <0.0001 |
| SAPS II score, median [IQR] | 2203 | 50.00 [38.00-63.00] | 41.00 [32.00-53.00] | 55.50 [44-65.25] | 51.00 [41.00-63.50] | 56.00 [46.00-69.00] | <0.0001 |
| Serum creatinine in mg/dL, median [IQR] | 2203 | 1.30 [0.81-2.28] | 0.76 [0.60-0.90] | 0.90 [0.80-1.00] | 1.55 [1.35-1.92] | 2.45 [1.80-3.44] | <0.0001 |
| penKid in pmol/L, median [IQR] | 2203 | 82.10 [48.37-152.30] | 45.03 [33.36-60.27] | 113.53 [100.22-144.21] | 64.82 [49.44-77.29] | 168.68 [122.85-243.16] | <0.0001 |
| WRF, no. (%) | 2203 | 644 (29.2) | 90 (11.1) | 38 (27.1) | 88 (24) | 428 (48.3) | <0.0001 |
| 28d mortality, no. (%) | 2203 | 552 (25.1) | 124 (15.3) | 54 (38.6) | 67 (18.3) | 307 (34.7) | <0.0001 |

Supplementary Table 4: Patient characteristics at baseline per study included in the patient-level meta-analysis.

|  | **AdrenOSS-1 (n=576, 26.1%)** | **ALBIOS (n=919, 41.7%)** | **FROG-ICU (n=512, 23.2%)** | **PredARRT-Sep (n=196, 8.9%)** | **P-value** |
| --- | --- | --- | --- | --- | --- |
| Age, median [IQR] | 65.64 [55.26-75.44] | 70 [58-78] | 67 [56-77] | 66 [59-74] | 0.0016 |
| Sex (male), no. (%) | 360 (62.5) | 539 (58.7) | 336 (65.6) | 127 (64.8) | 0.0481 |
| Body mass index in kg/m², median [IQR] | 25.71 [22.86-30.12] | 25.71 [23.41-28.41] | 27.47 [24.33-31.58] | 27.14 [24.22-31.01] | <0.0001 |
| Hypotension, no. (%) | 288 (73.5) | 93 (10.1) | 246 (48) | 141 (71.9) | <0.0001 |
| CKD, no. (%) | 75 (18.5) | 40 (4.4) | 76 (14.8) | 48 (24.5) | <0.0001 |
| RRT, no. (%) | 49 (8.5) | 78 (8.5) | 13 (2.5) | 9 (4.6) | <0.0001 |
| Sepsis, no. (%) | 285 (49.5) | 407 (44.3) | 46 (9) | 36 (18.4) | <0.0001 |
| Septic Shock, no. (%) | 291 (50.5) | 512 (55.7) | 466 (91) | 160 (81.6) | <0.0001 |
| SAPS II score, median [IQR] | 50 [40-63] | 46 [35-56] | 54 [40.00-65.25] | 67 [51-78] | <0.0001 |
| Serum creatinine in mg/dL, median [IQR] | 1.35 [0.87-2.21] | 1.3 [0.80-2.30] | 1.22 [0.79-2.18] | 1.47 [0.92-2.33] | 0.0566 |
| penKid in pmol/L, median [IQR] | 83.9 [52.68-154.73] | 82.79 [49.36-158.64] | 84.81 [46.02-156.5] | 73.82 [40.53-114.18] | 0.002 |

Supplementary Table 5: Number of patients per study grouped by the primary endpoint WRF and the secondary endpoint 28d-mortality.

| **Clinical studies** | **All (%)** | **No WRF (%)** | **WRF (%)** | **Alive (%)** | **Deceased (%)** |
| --- | --- | --- | --- | --- | --- |
| AdrenOSS-1, no. (%) | 576 (26.1) | 417 (72.4) | 159 (27.6) | 451 (78.3) | 125 (21.7) |
| ALBIOS, no. (%) | 919 (41.7) | 622 (67.7) | 297 (32.3) | 670 (72.9) | 249 (27.1) |
| FROG-ICU, no. (%) | 512 (23.2) | 384 (75.0) | 128 (25.0) | 375 (73.2) | 137 (26.8) |
| PredARRT, no. (%) | 196 (8.9) | 136 (69.4) | 60 (30.6) | 155 (79.1) | 41 (20.9) |

Supplementary Table 6: Number of patients per study grouped by normal and elevated sCr and penKid based on the clinical cut-off 89 pmol/L for penKid and a sex-specific
cut-off for sCr (1.22 mg/dL for males and 1.01 mg/dL for females).

| **Clinical studies** | **All (%)** | **Normal SCr, normal penKid** | **Normal SCr, elevated penKid** | **Elevated SCr, normal penKid** | **Elevated SCr, elevated penKid** |
| --- | --- | --- | --- | --- | --- |
| AdrenOSS-1, no. (%) | 576 (26.1) | 197 (34.2) | 32 (5.6) | 105 (18.2) | 242 (42) |
| ALBIOS, no. (%) | 919 (41.7) | 346 (37.6) | 57 (6.2) | 142 (15.5) | 374 (40.7) |
| FROG-ICU, no. (%) | 512 (23.2) | 196 (38.3) | 46 (9) | 68 (13.3) | 202 (39.5) |
| PredARRT, no. (%) | 196 (8.9) | 71 (36.2) | 5 (2.6) | 52 (26.5) | 68 (34.7) |

Supplementary Table 7A: Performance characteristics of penKid and sCr for the primary endpoint WRF. Sensitivity, specificity, PPV and NPV are based on the cut-off 89 pmol/L for penKid and the sex-specific cut-off (1.22 mg/dL for males and 1.01 mg/dL for females) for sCr. PPV – positive predictive value, NPV – negative predictive value, AUC – area under the curve, penKid – Proenkephalin A 119-159, sCr – serum creatinine.

|  | Sensitivity (95% CI) | Specificity (95% CI) | PPV (95% CI) | NPV (95% CI) | AUROC (95% CI) |
| --- | --- | --- | --- | --- | --- |
| penKid | 0.72 (0.69-0.76) | 0.64 (0.62-0.66) | 0.45 (0.42-0.48) | 0.85 (0.83-0.87) | 0.755 (0.732-0.777) |
| sCr | 0.80 (0.77-0.83) | 0.53 (0.50-0.55) | 0.41 (0.38-0.44) | 0.87 (0.84-0.89) | 0.722 (0.699-0.745) |

Supplementary Table 7B: Performance characteristics of penKid and sCr for the secondary endpoint 28d-mortality. Sensitivity, specificity, PPV and NPV are based on the cut-off 89 pmol/L for penKid and the sex-specific cut-off (1.22 mg/dL for males and 1.01 mg/dL for females) for sCr. PPV – positive predictive value, NPV – negative predictive value, AUC – area under the curve, penKid – Proenkephalin A 119-159, sCr – serum creatinine.

|  | Sensitivity (95% CI) | Specificity (95% CI) | PPV (95% CI) | NPV (95% CI) | AUROC (95% CI) |
| --- | --- | --- | --- | --- | --- |
| penKid | 0.65 (0.61-0.69) | 0.60 (0.57-0.62) | 0.35 (0.32-0.38) | 0.84 (0.82-0.86) | 0.660 (0.634-0.685) |
| sCr | 0.68 (0.64-0.72) | 0.47 (0.44-0.49) | 0.30 (0.27-0.32) | 0.81 (0.79-0.84) | 0.589 (0.563-0.616) |

Supplementary Table 8A: Performance characteristics of penKid in the subgroups normal- and elevated sCr for the primary endpoint WRF. Sensitivity, specificity, PPV and NPV are based on the cut-off 89 pmol/L for penKid and the sex-specific cut-off (1.22 mg/dL for males and 1.01 mg/dL for females) for sCr. PPV – positive predictive value, NPV – negative predictive value, AUC – area under the curve, penKid – Proenkephalin A 119-159, sCr – serum creatinine.

|  |  | Sensitivity (95% CI) | Specificity (95% CI) | PPV (95% CI) | NPV (95% CI) | AUROC (95% CI) |
| --- | --- | --- | --- | --- | --- | --- |
| penKid | Normal SCr (n=950) | 0.30 (0.22-0.38) | 0.88 (0.85-0.90) | 0.27 (0.20-0.35) | 0.89 (0.87-0.91) | 0.690 (0.642-0.738) |
|  | Elevated SCr (n=1253) | 0.83 (0.80-0.86) | 0.38 (0.34-0.41) | 0.48 (0.45-0.52) | 0.76 (0.72-0.80) | 0.696 (0.666-0.725) |

Supplementary Table 8B: Performance characteristics of penKid in the subgroups normal- and elevated sCr for the secondary endpoint 28d-mortality. Sensitivity, specificity, PPV and NPV are based on the cut-off 89 pmol/L for penKid and the sex-specific cut-off (1.22 mg/dL for males and 1.01 mg/dL for females) for sCr. PPV – positive predictive value, NPV – negative predictive value, AUC – area under the curve, penKid – Proenkephalin A 119-159, sCr – serum creatinine.

|  |  | Sensitivity (95% CI) | Specificity (95% CI) | PPV (95% CI) | NPV (95% CI) | AUROC (95% CI) |
| --- | --- | --- | --- | --- | --- | --- |
| penKid | Normal SCr (n=950) | 0.30 (0.24-0.37) | 0.89 (0.87-0.91) | 0.39 (0.31-0.47) | 0.85 (0.82-0.87) | 0.661 (0.616-0.706) |
|  | Elevated SCr (n=1253) | 0.82 (0.78-0.86) | 0.34 (0.31-0.37) | 0.35 (0.32-0.38) | 0.82 (0.78-0.86) | 0.624 (0.591-0.657) |

Supplementary Table 9: Literature appraisal indicating title and abstract screening and full-text screening including exclusion rationale.

| **Source** | **Title and abstract screening - Reason for exclusion** | **Full text screening -  Reason for exclusion** | **PMID** | **Title** | **Citation** |
| --- | --- | --- | --- | --- | --- |
| Pubmed | no primary data (review) | N/A | 39127297 | Persistent acute kidney injury biomarkers: A systematic review and meta-analysis | Shi K, Jiang W, Song L, Li X, Zhang C, Li L, Feng Y, Yang J, Wang T, Wang H, Zhou L, Yu J, Zheng R. Persistent acute kidney injury biomarkers: A systematic review and meta-analysis. Clin Chim Acta. 2025 Jan 1;564:119907. doi: 10.1016/j.cca.2024.119907. Epub 2024 Aug 8. PMID: 39127297. |
|  | no primary data (review) | N/A | 39767206 | Sepsis-Associated Acute Kidney Injury: What's New Regarding Its Diagnostics and Therapeutics? | Kounatidis D, Tzivaki I, Daskalopoulou S, Daskou A, Adamou A, Rigatou A, Sdogkos E, Karampela I, Dalamaga M, Vallianou NG. Sepsis-Associated Acute Kidney Injury: What's New Regarding Its Diagnostics and Therapeutics? Diagnostics (Basel). 2024 Dec 17;14(24):2845. doi: 10.3390/diagnostics14242845. PMID: 39767206; PMCID: PMC11674886. |
|  |  | ED setting | 38790966 | Point-of-Care Serum Proenkephalin as an Early Predictor of Mortality in Patients Presenting to the Emergency Department with Septic Shock | Verras C, Bezati S, Bistola V, Ventoulis I, Matsiras D, Tsiodras S, Parissis J, Polyzogopoulou E. Point-of-Care Serum Proenkephalin as an Early Predictor of Mortality in Patients Presenting to the Emergency Department with Septic Shock. Biomedicines. 2024 May 2;12(5):1004. doi: 10.3390/biomedicines12051004. PMID: 38790966; PMCID: PMC11117930. |
|  | no primary data (review) | N/A | 38541160 | Sepsis-Associated Acute Kidney Injury: Where Are We Now? | Kounatidis D, Vallianou NG, Psallida S, Panagopoulos F, Margellou E, Tsilingiris D, Karampela I, Stratigou T, Dalamaga M. Sepsis-Associated Acute Kidney Injury: Where Are We Now? Medicina (Kaunas). 2024 Mar 6;60(3):434. doi: 10.3390/medicina60030434. PMID: 38541160; PMCID: PMC10971830. |
|  | no primary data (review) | N/A | 38476861 | Prognostic Biomarkers and AKI: Potential to Enhance the Identification of Post-Operative Patients at Risk of Loss of Renal Function | Singh R, Watchorn JC, Zarbock A, Forni LG. Prognostic Biomarkers and AKI: Potential to Enhance the Identification of Post-Operative Patients at Risk of Loss of Renal Function. Res Rep Urol. 2024 Mar 5;16:65-78. doi: 10.2147/RRU.S385856. PMID: 38476861; PMCID: PMC10928916. |
|  |  | No baseline creatinine | 34278538 | Plasma proenkephalin A 119-159 on intensive care unit admission is a predictor of organ failure and 30-day mortality | Frigyesi A, Boström L, Lengquist M, Johnsson P, Lundberg OHM, Spångfors M, Annborn M, Cronberg T, Nielsen N, Levin H, Friberg H. Plasma proenkephalin A 119-159 on intensive care unit admission is a predictor of organ failure and 30-day mortality. Intensive Care Med Exp. 2021 Jul 19;9(1):36. doi: 10.1186/s40635-021-00396-6. PMID: 34278538; PMCID: PMC8286914. |
|  |  |  | 32516543 | Incidence and Outcome of Subclinical Acute Kidney Injury Using penKid in Critically Ill Patients | Dépret F, Hollinger A, Cariou A, Deye N, Vieillard-Baron A, Fournier MC, Jaber S, Damoisel C, Lu Q, Monnet X, Rennuit I, Darmon M, Leone M, Guidet B, Sonneville R, Montravers P, Pili-Floury S, Lefrant JY, Duranteau J, Laterre PF, Brechot N, Oueslati H, Cholley B, Struck J, Hartmann O, Mebazaa A, Gayat E, Legrand M. Incidence and Outcome of Subclinical Acute Kidney Injury Using penKid in Critically Ill Patients. Am J Respir Crit Care Med. 2020 Sep 15;202(6):822-829. doi: 10.1164/rccm.201910-1950OC. PMID: 32516543. |
|  | no sepsis | N/A | 31987659 | PenKid measurement at admission is associated with outcome in severely ill burn patients | Dépret F, Polina A, Amzallag J, Fayolle-Pivot L, Coutrot M, Chaussard M, Struck J, Hartmann O, Jully M, Fratani A, Oueslati H, Cupaciu A, Soussi S, Benyamina M, Guillemet L, Mebazaa A, Textoris J, Legrand M; PRONOBURN group. PenKid measurement at admission is associated with outcome in severely ill burn patients. Burns. 2020 Sep;46(6):1302-1309. doi: 10.1016/j.burns.2020.01.002. Epub 2020 Jan 25. PMID: 31987659. |
|  | cohort <100 patients | N/A | 31977957 | Proenkephalin Compared to Conventional Methods to Assess Kidney Function in Critically Ill Sepsis Patients | Beunders R, van Groenendael R, Leijte GP, Kox M, Pickkers P. Proenkephalin Compared to Conventional Methods to Assess Kidney Function in Critically Ill Sepsis Patients. Shock. 2020 Sep;54(3):308-314. doi: 10.1097/SHK.0000000000001510. PMID: 31977957; PMCID: PMC7458088. |
|  |  | ED setting | 31779591 | Proenkephalin a 119-159 (penKid) - a novel biomarker for acute kidney injury in sepsis: an observational study | Rosenqvist M, Bronton K, Hartmann O, Bergmann A, Struck J, Melander O. Proenkephalin a 119-159 (penKid) - a novel biomarker for acute kidney injury in sepsis: an observational study. BMC Emerg Med. 2019 Nov 28;19(1):75. doi: 10.1186/s12873-019-0283-9. PMID: 31779591; PMCID: PMC6883703. |
|  |  |  | 30450469 | Proenkephalin A 119-159 (Penkid) Is an Early Biomarker of Septic Acute Kidney Injury: The Kidney in Sepsis and Septic Shock (Kid-SSS) Study | Hollinger A, Wittebole X, François B, Pickkers P, Antonelli M, Gayat E, Chousterman BG, Lascarrou JB, Dugernier T, Di Somma S, Struck J, Bergmann A, Beishuizen A, Constantin JM, Damoisel C, Deye N, Gaudry S, Huberlant V, Marx G, Mercier E, Oueslati H, Hartmann O, Sonneville R, Laterre PF, Mebazaa A, Legrand M. Proenkephalin A 119-159 (Penkid) Is an Early Biomarker of Septic Acute Kidney Injury: The Kidney in Sepsis and Septic Shock (Kid-SSS) Study. Kidney Int Rep. 2018 Aug 22;3(6):1424-1433. doi: 10.1016/j.ekir.2018.08.006. Erratum in: Kidney Int Rep. 2018 Nov 17;4(1):187. doi: 10.1016/j.ekir.2018.11.006. PMID: 30450469; PMCID: PMC6224621. |
|  |  |  | 30012641 | Circulating Proenkephalin, Acute Kidney Injury, and Its Improvement in Patients with Severe Sepsis or Shock | Caironi P, Latini R, Struck J, Hartmann O, Bergmann A, Bellato V, Ferraris S, Tognoni G, Pesenti A, Gattinoni L, Masson S; ALBIOS Study Investigators. Circulating Proenkephalin, Acute Kidney Injury, and Its Improvement in Patients with Severe Sepsis or Shock. Clin Chem. 2018 Sep;64(9):1361-1369. doi: 10.1373/clinchem.2018.288068. Epub 2018 Jul 16. PMID: 30012641. |
|  | no primary data (review) | N/A | 33636859 | Proenkephalin (PENK) as a Novel Biomarker for Kidney Function | Beunders R, Struck J, Wu AHB, Zarbock A, Di Somma S, Mehta RL, Koyner JL, Nadim MK, Maisel AS, Murray PT, Neath SX, Jaffe A, Pickkers P. Proenkephalin (PENK) as a Novel Biomarker for Kidney Function. J Appl Lab Med. 2017 Nov 1;2(3):400-412. doi: 10.1373/jalm.2017.023598. PMID: 33636859. |
|  |  | ED setting | 25486879 | Diagnostic and short-term prognostic utility of plasma pro-enkephalin (pro-ENK) for acute kidney injury in patients admitted with sepsis in the emergency department | Marino R, Struck J, Hartmann O, Maisel AS, Rehfeldt M, Magrini L, Melander O, Bergmann A, Di Somma S. Diagnostic and short-term prognostic utility of plasma pro-enkephalin (pro-ENK) for acute kidney injury in patients admitted with sepsis in the emergency department. J Nephrol. 2015 Dec;28(6):717-24. doi: 10.1007/s40620-014-0163-z. Epub 2014 Dec 9. PMID: 25486879. |
|  | no penKid data | N/A | 29265612 | Special pharmacokinetics of fluconazole in septic, obese and burn patients | Pittrow L, Penk A. Special pharmacokinetics of fluconazole in septic, obese and burn patients. Mycoses. 1999 Dec;42 Suppl 2:87-90. doi: 10.1111/j.1439-0507.1999.tb00020.x. PMID: 29265612. |
|  | duplicate | N/A | 10865911 | Special pharmacokinetics of fluconazole in septic, obese and burn patients | Pittrow L, Penk A. Special pharmacokinetics of fluconazole in septic, obese and burn patients. Mycoses. 1999;42 Suppl 2:87-90. PMID: 10865911. |
| Conference proceedings |  |  | 31584458 | Cell Cycle Biomarkers and Soluble Urokinase-Type Plasminogen Activator Receptor for the Prediction of Sepsis-Induced Acute Kidney Injury Requiring Renal Replacement Therapy: A Prospective, Exploratory Study. | Nusshag C, Rupp C, Schmitt F, Krautkrämer E, Speer C, Kälble F, Tamulyte S, Bruckner T, Zeier M, Reiser J, Weigand MA, Uhle F, Merle U, Morath C, Brenner T. Cell Cycle Biomarkers and Soluble Urokinase-Type Plasminogen Activator Receptor for the Prediction of Sepsis-Induced Acute Kidney Injury Requiring Renal Replacement Therapy: A Prospective, Exploratory Study. Crit Care Med. 2019 Dec;47(12):e999-e1007. doi: 10.1097/CCM.0000000000004042. PMID: 31584458; PMCID: PMC6867703. |

**References**

1. Levy M, Fink M, Marshall J, Abraham E, Angus D, Cook D, et al. 2001 SCCM/ESICM/ACCP/ATS/SIS International Sepsis Definitions Conference Critical care medicine. 2003;31(4):1250-6.

2. Mebazaa A, Geven C, Hollinger A, Wittebole X, Chousterman BG, Blet A, et al. Circulating adrenomedullin estimates survival and reversibility of organ failure in sepsis: the prospective observational multinational Adrenomedullin and Outcome in Sepsis and Septic Shock-1 (AdrenOSS-1) study. Critical care. 2018;22(1):354.

3. Mebazaa A, Casadio MC, Azoulay E, Guidet B, Jaber S, Levy B, et al. Post-ICU discharge and outcome: rationale and methods of the The French and euRopean Outcome reGistry in Intensive Care Units (FROG-ICU) observational study. BMC anesthesiology. 2015;15:143.

4. Bone RC, Balk RA, Cerra FB, Dellinger RP, Fein AM, Knaus WA, et al. Definitions for sepsis and organ failure and guidelines for the use of innovative therapies in sepsis. The ACCP/SCCM Consensus Conference Committee. American College of Chest Physicians/Society of Critical Care Medicine. Chest. 1992;101(6):1644-55.

5. Caironi P, Latini R, Struck J, Hartmann O, Bergmann A, Bellato V, et al. Circulating Proenkephalin, Acute Kidney Injury, and Its Improvement in Patients with Severe Sepsis or Shock. Clinical chemistry. 2018;64(9):1361-9.

6. Caironi P, Tognoni G, Masson S, Fumagalli R, Pesenti A, Romero M, et al. Albumin replacement in patients with severe sepsis or septic shock. The New England journal of medicine. 2014;370(15):1412-21.

7. Evans L, Rhodes A, Alhazzani W, Antonelli M, Coopersmith CM, French C, et al. Surviving Sepsis Campaign: International Guidelines for Management of Sepsis and Septic Shock 2021. Critical care medicine. 2021;49(11):e1063-e143.

8. Nusshag C, Rupp C, Schmitt F, Krautkrämer E, Speer C, Kälble F, et al. Cell Cycle Biomarkers and Soluble Urokinase-Type Plasminogen Activator Receptor for the Prediction of Sepsis-Induced Acute Kidney Injury Requiring Renal Replacement Therapy: A Prospective, Exploratory Study. Critical care medicine. 2019;47(12):e999-e1007.

9. Tavris BS, Morath C, Rupp C, Szudarek R, Uhle F, Sweeney TE, et al. Complementary role of transcriptomic endotyping and protein-based biomarkers for risk stratification in sepsis-associated acute kidney injury. Critical care. 2025;29(1):136.
