# Supplementary material for: Clinical utility of proenkephalin A 119-159 for prediction of worsening renal function and prognosis in patients with sepsis –results of a patient-level meta-analysis
Source: Crit Care. 2026 Mar 24;30:164. doi: 10.1186/s13054-026-05947-5 (PMC13064392; doi:10.1186/s13054-026-05947-5)
Supplement: Supplementary file 2 — Supplementary Material 2 [file 13054_2026_5947_MOESM2_ESM.pdf]

Supplementary Figures

Supplementary Figure 1: PRISMA flow diagram
Supplementary Figure 2: Flow diagram of the patient population analyzed in this patient-level meta-analysis.
Supplementary Figure 3: Twenty-eight day Kaplan Meier survival curves and and cox proportional hazard regression analysis comparing normal- and elevated levels of penKid and sCr.
Supplementary Figure 4: Prediction of endpoints WRF and 28d mortality using penKid or serum creatinine. Receiver-operating characteristics analysis for penKid or sCr for WRF and 28d-mortality.
Supplementary Figure 5: Receiver-operating characteristics analysis for sCr and penKid in the subgroups normal- and elevated sCr for the primary endpoint WRF and the secondary endpoint 28d-mortality.

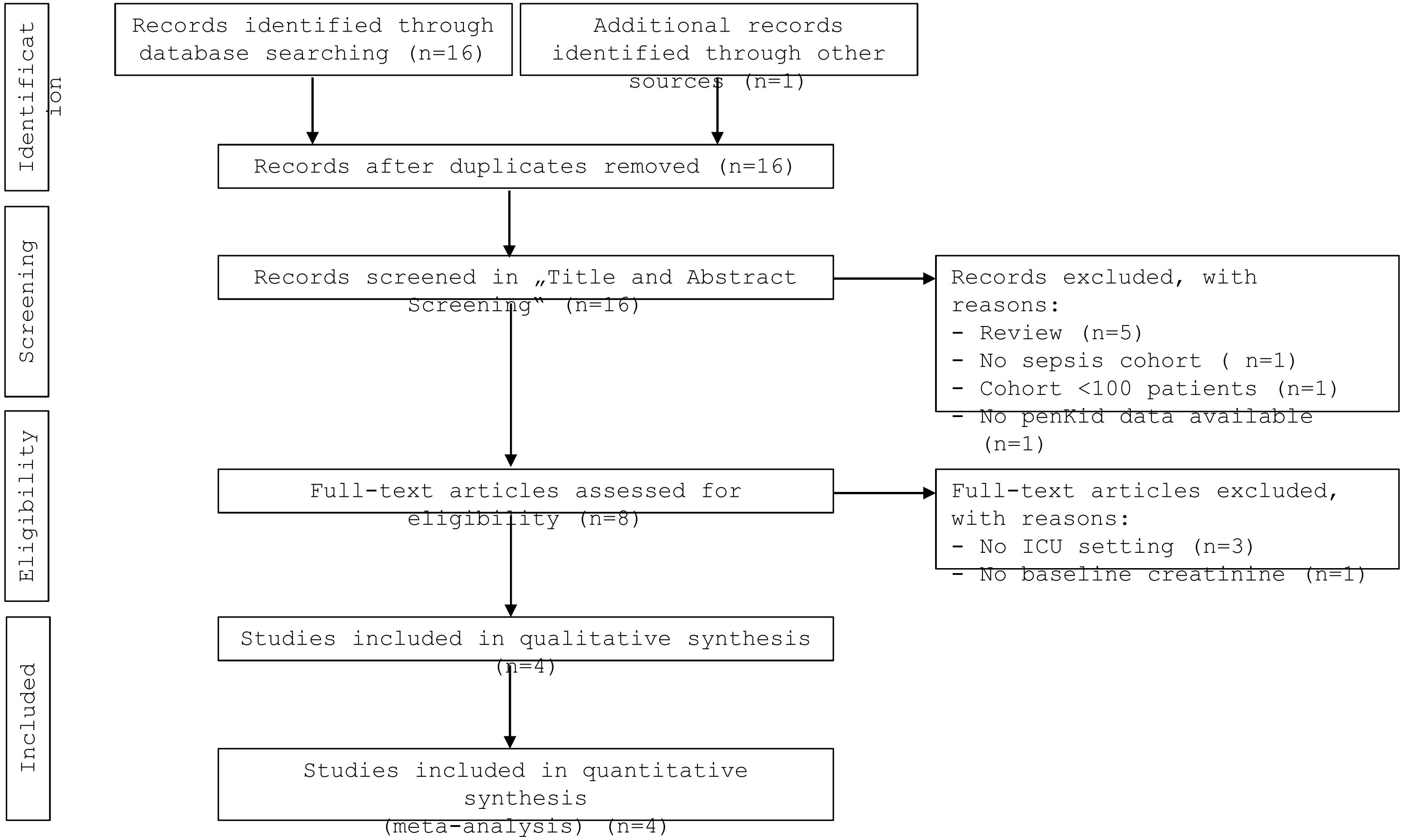

Supplementary Figure 1: PRISMA flow diagram illustrating the study selection process for the patient-level meta-analysis. The flowchart details the number of records identified through database searching and other sources, the number of records screened, full-text articles assessed for eligibility, and studies included in the final analysis. Reasons for exclusion at each stage are also indicated.

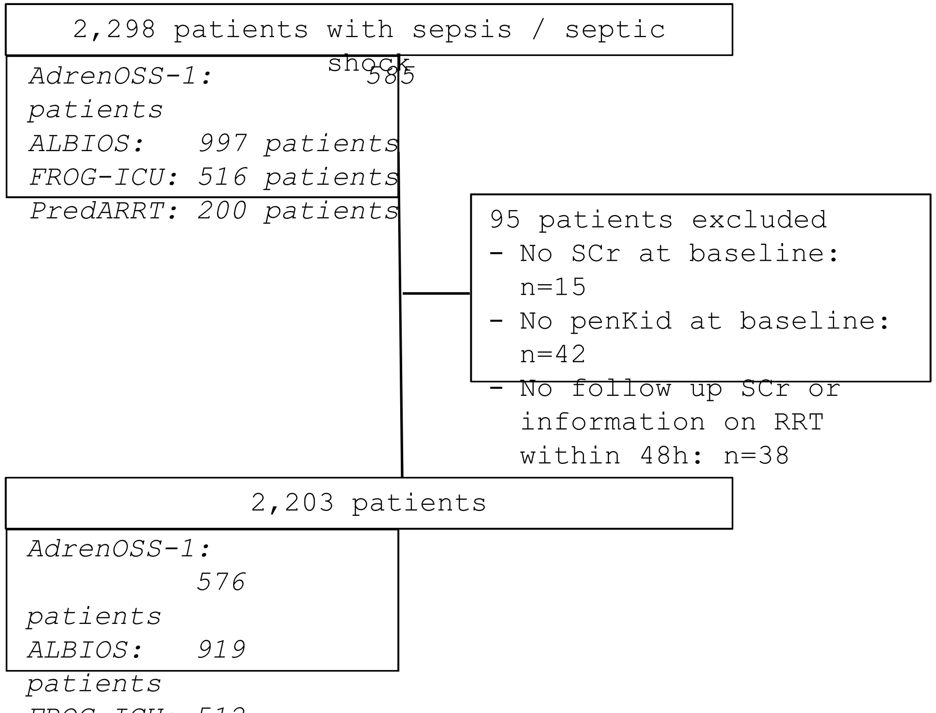

Supplementary Figure 2: Flow diagram of the patient population analyzed in this patient-level meta-analysis.

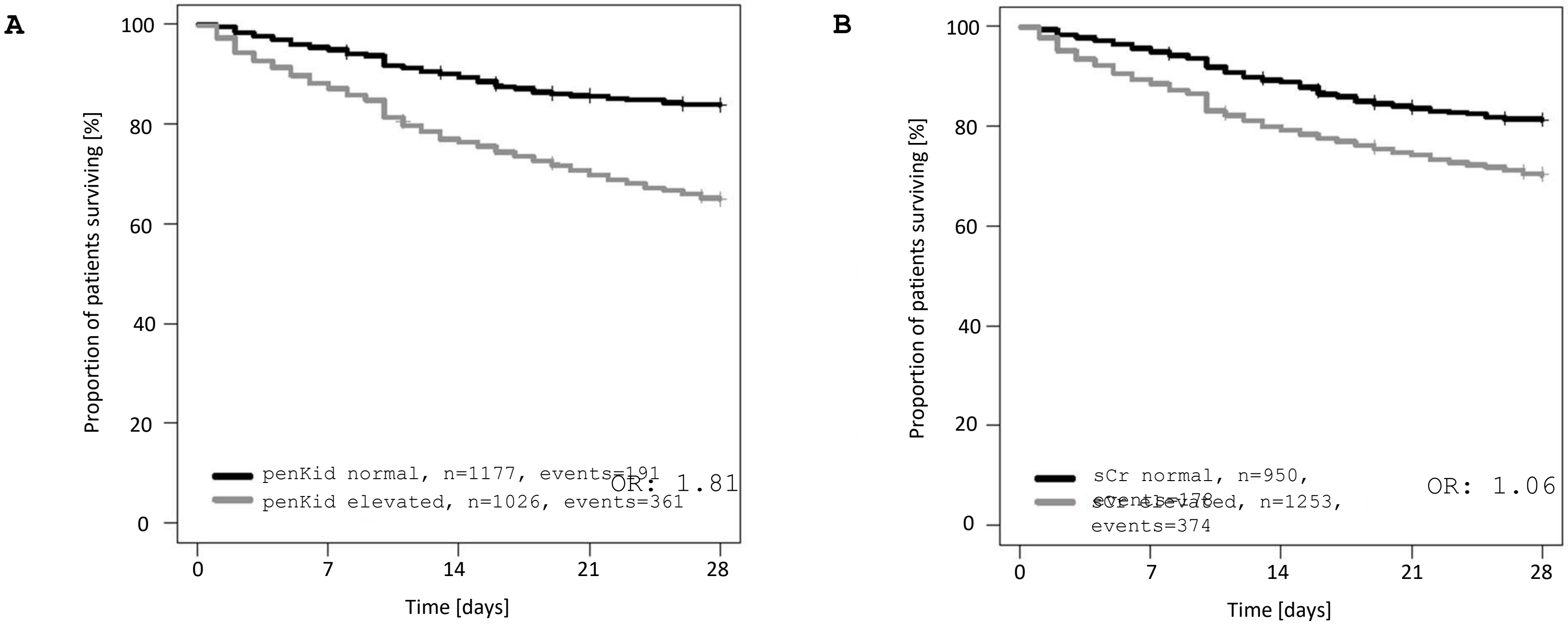

Supplementary Figure 3: Twenty-eight day Kaplan Meier survival curves and and logistic regression analysis comparing normal- and elevated levels of penKid (A) and sCr (B) based on the clinical cut-off 89 pmol/L for penKid and a sex-specific cut-off for sCr (1.22 mg/dL for males and 1.01 mg/dL for females). penKid – Proenkephalin A 119-159, sCr – serum creatinine.

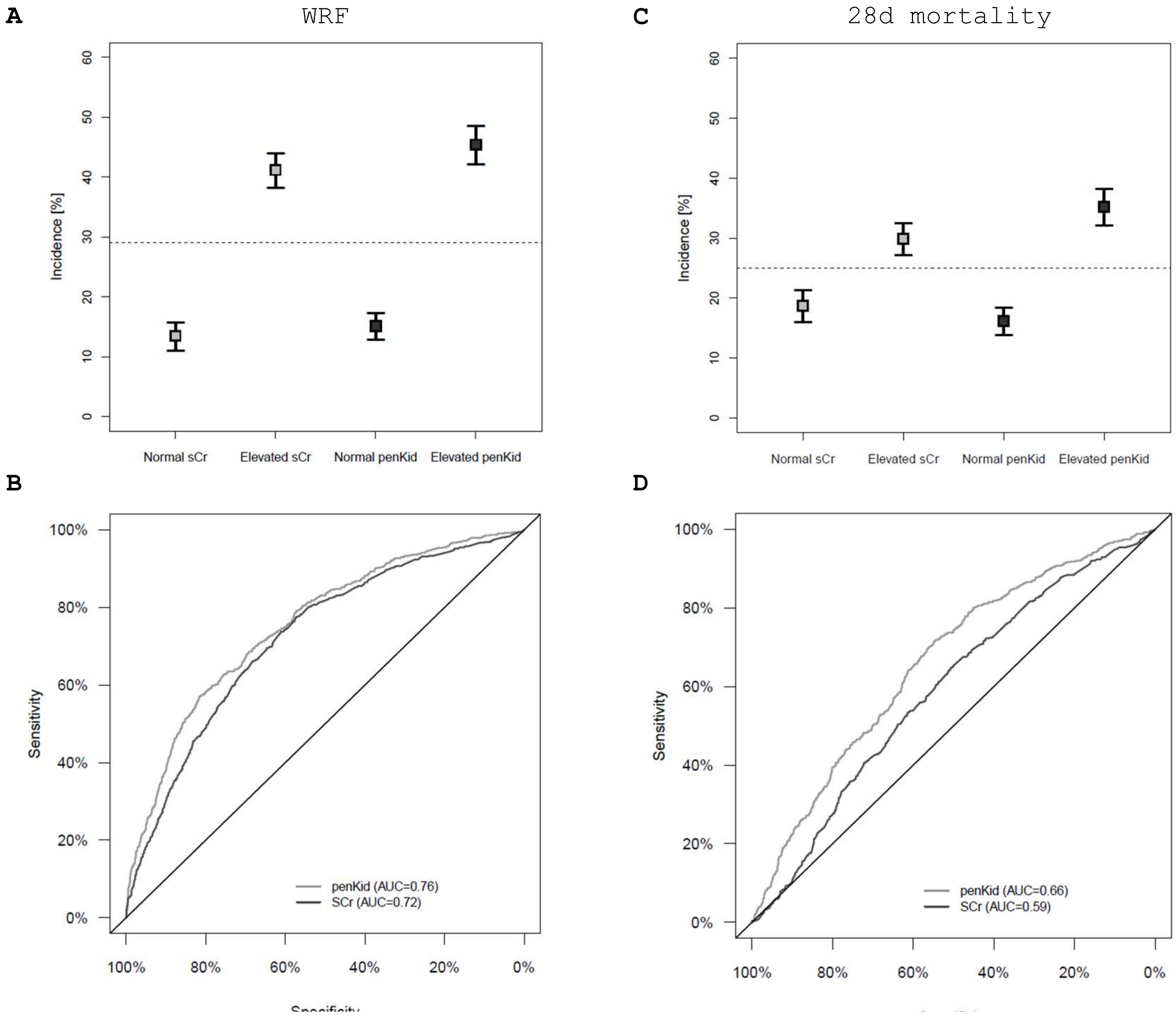

Supplementary Figure 4: Prediction of endpoints WRF and 28d mortality using penKid or serum creatinine. Cut-off derived incidence rates for penKid and sCr (89 pmol/L for penKid, gender-specific cut-off for sCr of 1.22 mg/dL for males and 1.01 mg/dL for females) for the primary endpoint WRF (A) and the secondary endpoint 28d-mortality (C). Dashed line represents the global pretest probability (WRF: 29.2% n=644, 28d-mortality: 25.1% n=552). Receiver-operating characteristics analysis for penKid or sCr for WRF (B) and 28d-mortality (D). penKid – Proenkephalin A 119-159, sCr – serum creatinine.

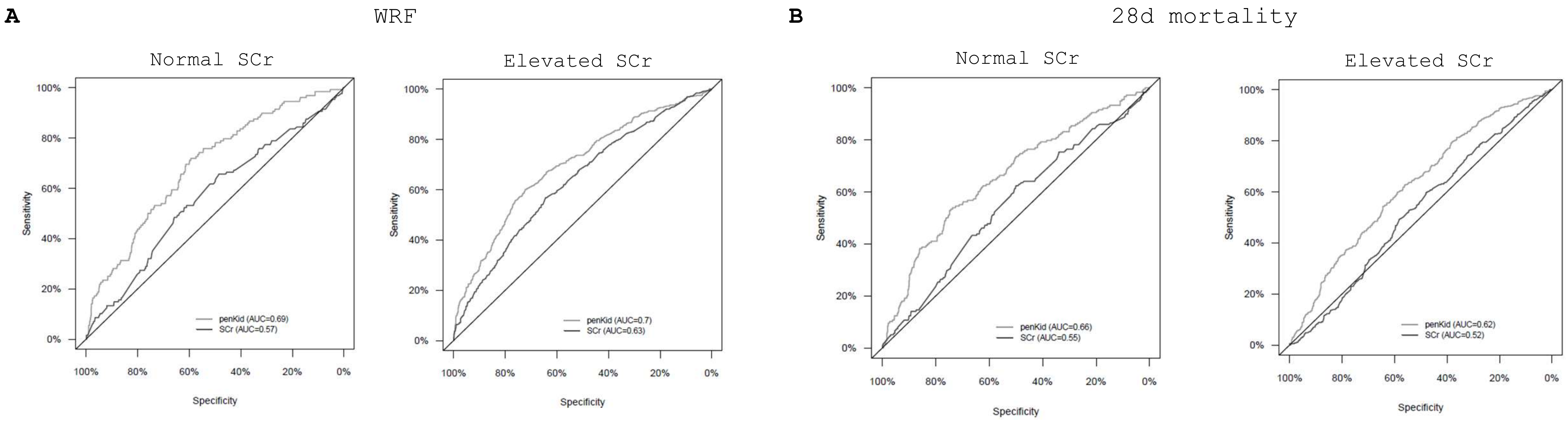

Supplementary Figure 5: Receiver-operating characteristics analysis for sCr and penKid in the subgroups normal- and elevated sCr (based on the sex-specific cut-off for sCr 1.22 mg/dL for males and 1.01 mg/dL for females) for the primary endpoint WRF (A) and the secondary endpoint 28d-mortality (B). AUC – area under the curve, penKid – Proenkephalin A 119-159, sCr – serum creatinine.
