# Supplementary material for: Clinical utility of proenkephalin A 119-159 for prediction of worsening renal function and prognosis in patients with sepsis –results of a patient-level meta-analysis
Source: Crit Care. 2026 Mar 24;30:164. doi: 10.1186/s13054-026-05947-5 (PMC13064392; doi:10.1186/s13054-026-05947-5)
Supplement: Supplementary file 3 — Supplementary Material 3 [file 13054_2026_5947_MOESM3_ESM.docx]

**Supplementary Methods**

Search strategy

To identify studies eligible for this systematic analysis, the database PubMed (search terms “sepsis AND (penKid OR proenkephalin OR PENK) AND creatinine”) and conference proceedings have been searched independently by two reviewers (BA and FU). The search was conducted till March 20, 2025. We excluded reviews, abstracts without full text, duplicate publications and studies with no available baseline data for penKid or sCr. Studies were assessed initially by title and abstract screening and then by full-text screening (Suppl. Table 9). The two reviewers collected the data independently from each other.

penKid measurements

Proenkephalin A 119-159 (penKid) has been assessed at ICU admission in EDTA plasma samples of all four studies using the immunoluminometric assay sphingotest® penKid® (SphingoTec GmbH, Hennigsdorf, Germany) as described previously (1). The laboratory performing the biomarker measurement was blinded to clinical and demographic data of the patients. Based on the manufacturer’s instruction for use, the 97.5^th^ percentile for sphingotest® penKid® in healthy adult subjects is 89 pmol/L (90% CI 85 – 118 pmol/L). The upper normal range (89 pmol/L) is also the established clinical cut-off for diagnosis of acute kidney injury (2).

Risk of bias assessment

Risk of bias in individual studies included in this meta-analysis have been assessed using the QUADAS-2 (Quality Assessment of Diagnostic Accuracy Studies) tool, including the following domains: (i) patient selection; (ii) index test; (iii) reference standard; (iv) flow and timing. Two independent reviewers (BA and FU) have assessed the risk of bias for each study. Disagreements have been resolved by a third reviewer (OH) to reach a final assessment. The risk has been categorized as low, high, or unclear for each domain.

Statistical methods

Group comparisons of continuous variables were performed using the Kruskal-Wallis test. Biomarker data were log-transformed if necessary. Categorical data were compared using Pearson's Chi-squared Test for Count Data.

Receiver-operating-characteristic (ROC) curves were constructed to assess the sensitivity and specificity, and the area under the receiver operating characteristic curve (AUC_ROC_) was calculated. Sensitivity, specificity, positive predictive values (PPV) and negative predictive values (NPV) were determined based on the established clinical cut-offs for the diagnosis of AKI; for penKid the predefined cut-off at 89 pmol/L was applied (2); for sCr, a sex-specific cut point - 1.22 mg/dL for males and 1.01 mg/dL for females - was applied (3).

All statistical tests were 2-tailed and a two-sided p-value of <0.05 was considered for significance. The statistical analyses were performed using R version 4.2.2 (http://www.r-project.org, library rms, Hmisc, ROCR) and Statistical Package for the Social Sciences (SPSS) version 22.0 (SPSS Inc., Chicago, Illinois, USA).

References

1. Donato LJ, Meeusen JW, Lieske JC, Bergmann D, Sparwasser A, Jaffe AS. Analytical performance of an immunoassay to measure proenkephalin. Clinical biochemistry. 2018;58:72-7.

2. Schulte J, Dépret F, Hartmann O, Pickkers P, Laterre P-F, Uhle F, et al. Clinical performance of proenkephalin A 119-159 for the early diagnosis of acute kidney injury in patients with sepsis or septic shock. medRxiv preprint. 2024.

3. Hannemann A, Friedrich N, Dittmann K, Spielhagen C, Wallaschofski H, Völzke H, et al. Age- and sex-specific reference limits for creatinine, cystatin C and the estimated glomerular filtration rate. Clinical chemistry and laboratory medicine : CCLM / FESCC. 2011;50(5):919-26.
